# Exfoliation Resistance, Microstructure, and Oxide Formation Mechanisms of the White Oxide Layer on CP Ti and Ti–Nb–Ta–Zr Alloys

**DOI:** 10.3390/ma14216599

**Published:** 2021-11-02

**Authors:** Eri Miura-Fujiwara, Soichiro Yamada, Keisuke Mizushima, Masahiko Nishijima, Yoshimi Watanabe, Toshihiro Kasuga, Mitsuo Niinomi

**Affiliations:** 1 Graduate School of Engineering, University of Hyogo, 2167 Shosha, Himeji, Hyogo 671-2280, Japan; cub_win_nurse@yahoo.co.jp; 2 Graduate School of Engineering, Nagoya Institute of Technology, Gokisocho, Showa Ward, Nagoya, Aichi 466-8555, Japan; cgz13172@yahoo.co.jp (S.Y.); yoshimi@nitech.ac.jp (Y.W.); kasuga.toshihiro@nitech.ac.jp (T.K.); 3 Institute for Protein Research, Osaka University 3-2 Yamadaoka, Suita, Osaka 565-0871, Japan; m.y.nishijima@protein.osaka-u.ac.jp (M.N.); niinomi@imr.tohoku.ac.jp (M.N.); 4 Graduate School of Engineering, Meijo University, 1-501 Shiogamaguchi, Tenpaku Ward, Nagoya, Aichi 468-8502, Japan; 5 Graduate School of Engineering, Osaka University, 2-1 Yamadaoka, Suita, Osaka 565-0871, Japan

**Keywords:** titanium–niobium–tantalum–zirconium (Ti–Nb–Ta–Zr) alloy, oxide coating, biomaterials, exfoliation resistance, interfacial microstructure, nanoindentation, fretting wear

## Abstract

We found that specific biomedical Ti and its alloys, such as CP Ti, Ti–29Nb–13Ta–4.6Zr, and Ti–36Nb–2Ta–3Zr–0.3O, form a bright white oxide layer after a particular oxidation heat treatment. In this paper, the interfacial microstructure of the oxide layer on Ti–29Nb–13Ta–4.6Zr and the exfoliation resistance of commercially pure (CP) Ti, Ti–29Nb–13Ta–4.6Zr, and Ti–36Nb–2Ta–3Zr–0.3O were investigated. The alloys investigated were oxidized at 1273 or 1323 K for 0.3–3.6 ks in an air furnace. The exfoliation stress of the oxide layer was high in Ti–29Nb–13Ta–4.6Zr and Ti–36Nb–2Ta–3Zr–0.3O, and the maximum exfoliation stress was as high as 70 MPa, which is almost the same as the stress exhibited by epoxy adhesives, whereas the exfoliation stress of the oxide layer on CP Ti was less than 7 MPa, regardless of duration time. The nanoindentation hardness and frictional coefficients of the oxide layer on Ti–29Nb–13Ta–4.6Zr suggested that the oxide layer was hard and robust enough for artificial tooth coating. The cross-sectional transmission electron microscopic observations of the microstructure of oxidized Ti–29Nb–13Ta–4.6Zr revealed that a continuous oxide layer formed on the surface of the alloys. The Au marker method revealed that both in- and out-diffusion occur during oxidation in Ti–29Nb–13Ta–4.6Zr and Ti–36Nb–2Ta–3Zr–0.3O, whereas only out-diffusion governs oxidation in CP Ti. The obtained results indicate that the high exfoliation resistance of the oxide layer on Ti–29Nb–13Ta–4.6Zr and Ti-36Nb-2Ta-3Zr-0.3O are attributed to their dense microstructures composing of fine particles, and a composition-graded interfacial microstructure. On the basis of the results of our microstructural observations, the oxide formation mechanism of the Ti–Nb–Ta–Zr alloy is discussed.

## 1. Introduction

Recently, the development of regenerative medicine has gained increasing attention and high expectations. Inorganic and organic biomedical materials still play important roles in the medical field; in particular, many such materials are used in dental prostheses. Thus, new alloys and surface treatments that aim to improve biocompatibility and/or osseointegration are frequently proposed. In this field, white-colored materials with mechanical properties similar to those of metal, i.e., “white metals” have long been sought after. The shade of color on an artificial tooth or orthodontic device is an important property, as are the tooth or device’s corrosion resistance and mechanical properties [1,2]. The simplest method of obtaining white-colored metals is to coat the metal with a white-colored material. However, the issue of exfoliation resistance has always been a problem with such hybrid materials.

Miura-Fujiwara et al. previously reported that a thick, yellowish-white oxide layer with high brightness formed on commercially pure titanium (CP Ti), Ti–29Nb–13Ta–4.6Zr, and Ti–36Nb–2Ta–3Zr–0.3O after they were heat-treated in air [3,4,5,6]. With respect to substrate metals, Ti–29Nb–13Ta–4.6Zr was developed by Kuroda and Niinomi et al. [7] as a biomedical β-type Ti alloy. This alloy was developed via molecular orbital calculations of electronic structures (DV-Xα cluster method) [8,9] to obtain the β-structure with a low Young’s modulus. Ti–36Nb–2Ta–3Zr–0.3O is among the series of Gummetal^®®^ substrates developed by Toyota Research Laboratory Co., Ltd. This alloy system exhibits dislocation-free plastic deformation, and some of the component alloys exhibit super plasticity [10,11,12,13,14]. Thus, Gummetal^®^ is already sold as a bracing wire in Japan [15].

It has been proposed that an ideal white metal could be prepared using Ti–29Nb–13Ta–4.6Zr and Ti–36Nb–2Ta–3Zr–0.3O if an esthetically and mechanically excellent oxide layer with durability against exfoliation can be prepared [3]. Reportedly, the oxide layers formed on CP Ti and Ti–29Nb–13Ta–4.6Zr determine their surface color tone, and investigations have revealed the relationships among color tone, oxide layer thickness, and heat-treatment conditions [3,4]; a manuscript detailing the properties of the oxide layer on Ti–36Nb–2Ta–3Zr–0.3O was also reported [6]. Obata et al. [5] investigated the cell compatibility of Ti–29Nb–13Ta–4.6Zr and oxide-coated Ti–29Nb–13Ta–4.6Zr and concluded that they exhibit almost the same compatibility and are suitable for biomedical use.

With respect to the color tone of the oxide layers on CP Ti, Ti–29Nb–13Ta–4.6Zr, and Ti–36Nb–2Ta–3Zr–0.3O, it has been reported that the brightness L* increases with increasing oxide layer thickness [4,6]. X-ray diffraction (XRD) investigations suggested that the oxide on CP Ti consisted of rutile-structured TiO_2_, whereas that on Ti–29Nb–13Ta–4.6Zr consisted of TiO_2_ and TiNb_2_O_7_ (or possibly Ti(Nb, Ta)_2_O_7_). Cross-sectional observations using scanning electron microscopy (SEM) revealed that CP Ti exhibits a “piecrust-like” layered structure consisting of stacked TiO_2_ layers and a gap a few microns thick [4]. SEM observations also revealed that a firm and robust oxide continuously formed on Ti–29Nb–13Ta–4.6Zr [4] and Ti–36Nb–2Ta–3Zr–0.3O [6] substrates. These results suggest that oxide layers on Ti–29Nb–13Ta–4.6Zr and Ti–36Nb–2Ta–3Zr–0.3O alloys can be used for dental devices when their exfoliation resistance is sufficiently high. The exfoliation resistance of the oxide layer on Ti–29Nb–13Ta–4.6Zr has been investigated and reported to be very high [4], whereas the oxide layer on Ti–36Nb–2Ta–3Zr–0.3O, which is included in the same Ti–Nb–Ta–Zr alloy system, has not yet been investigated. In general, although the oxide formation mechanisms are not yet understood, the microstructures of the oxide layer, substrate, and their interface are closely associated with the mechanical properties of the oxide-coated alloys.

Therefore, in this study, relationships among the cross-exfoliation resistance, heat-treatment conditions, and microstructure cross-sections of oxide layers on CP Ti, Ti–29Nb–13Ta–4.6Zr, and Ti–36Nb–2Ta–3Zr–0.3O were investigated. Additionally, the oxides’ friction coefficient measured under fretting conditions was studied to estimate their potential application as a surface coating for an artificial tooth. Furthermore, to determine whether the oxidation direction represents in- or out-diffusion, the Au marker method was employed. The Au marker method determines the major atomic migration direction via the insertion of a marker consisting of a nonreactive material that has little effect on the oxidation reaction itself. Since Au is hardly oxidized and has little reaction with Ti, Miura et al. used this method to clarify the reaction during Ti/porcelain firing [16,17]. It was discovered that the reaction layer formed on the porcelain side of the Au marker, and they concluded that Ti diffused into the porcelain [16,17]. We conducted similar experiments with CP Ti, Ti–29Nb–13Ta–4.6Zr, and Ti–36Nb–2Ta–3Zr–0.3O. Then, on the basis of the results obtained, we discussed their oxidation mechanism. New discussions, in addition to those on the preliminary results of the research on CP Ti and Ti–29Nb–13Ta–4.6Zr, which were previously reported [3,4,5,6], are presented in this study.

## 2. Materials and Methods

Hot-rolled Ti–36Nb–2Ta–3Zr–0.3O (Gummetal^®®^, Toyota Tsusho Material Inc., Nagoya, Japan), Ti–29Nb–13Ta–4.6Zr (TNTZ), and CP Ti (grade 2, Selec, Osaka, Japan) rods with diameters of 10–15 mm were used as the substrate material. The matrix phase of Gummetal and TNTZ is mainly composed of β phase with bcc structure at room temperature, while CP Ti is single α phase with hcp structure. The rods were sliced into discs with a thickness, *t*, of approximately *t* = 1 mm using a wire electron discharge machine. The discs were then polished with emery papers with grits up to #4000. The polished samples were oxidized in an air furnace at 1273 or 1323 K for 0.3–3.6 ks. The samples were furnace-cooled after being heat-treated.

The Au marker specimen was prepared as follows: after the sliced discs were mirror-polished using colloidal silica slurry, half of the surface of the disc was masked with masking tape. The Au coating was deposited using a magnetron sputtering system. The thickness of the Au coating was approximately 60 nm according to the catalog. After the masking tape was removed, the half-Au-coated discs were degreased by acetone. The specimens were oxidized in an air furnace at 1273 K for 1.8 ks.

The exfoliation resistance of the oxidized layers was measured using an adherence tester (ROMULUS, Quad Group Inc., Spokane, WA, USA). The schematic of the tester is presented in a previous study [4]. A stud pin with a diameter of 2.7 mm was glued onto the oxide layer side of each specimen using epoxy adhesives, and the stud pin with the specimen was subsequently placed onto the instrument’s retainer. The pin was fixed to the pull rod so that the pin was pulled with a constant force rate of $\dot{F}$ = 102.97 N/s. The exfoliation stress *σ*_e_ was obtained by *σ*_e_ = *F_m_*/*A*, where *F_m_* is the maximum exfoliation strength and *A* is the bonded area.

The nanoindentation hardness of the oxidized Ti–29Nb–13Ta–4.6Zr interface between the oxide and substrate was measured (RNT1100, Elionix, Tokyo, Japan). The oxidation conditions at 1273 K for 1.8 ks in air. A load-unload curve was recorded every 3 μm in the oxide layer and every 15 μm in the substrate along the length and depth of the alloy. Measurements were performed under 49 × 10^−3^ N for 0.5 μm, and the Vickers converted indentation hardness, *H*_IT_, was obtained. *H*_IT_ and HV have a linear relationship described using a coefficient, *C*_1_, as *H*_IT_ = *C*_1_ HV. The *C*_1_ value is approximately 1.25, which was obtained experimentally [18].

Fretting frictional tests were conducted using a fretting test machine with a reciprocal piezoelectric control stage (TYPE-30FS, Heidon, Tokyo, Japan). The sliding distance was approximately 100 μm, and the sliding frequency was 20 Hz. Tests were performed in Hanks’ balanced salt solution (HBSS, H8264, Sigma–Aldrich, Gillingham, UK) at 310 K, under 100 gf and 200 gf dead loads, and the counterface was a polished *φ*4.76 mm ZrO_2_ ball. Both a static frictional coefficient *μ_s_* and a dynamic frictional coefficient *μ*_d_ were obtained. The oxidized specimens evaluated in the nanoindentation and fretting tests were prepared under the oxidation conditions at 1273 K for 1.8 ks in air.

The microstructures were observed using an optical microscope (OM, BX41M-ESD, Olympus, Tokyo, Japan). Microstructure cross-sections were observed using an SEM with an energy dispersive X-ray spectroscope (SEM-EDS, JEM-7001FA, JEOL, Tokyo, Japan), a 200 kV scanning-transmission electron microscope (STEM, JEM-ARM200F, JEOL, Tokyo, Japan), and a 400 kV transmission electron microscope (TEM, JEM-2100F, JEM2010, JEOL, Tokyo, Japan) with EDS. Sample cross-sections for SEM were prepared by cutting an oxidized specimen embedded in resin perpendicular to the surface using a diamond wheel saw, and the cross-section was subsequently polished with colloidal silica. The oxide layer thickness was measured from the SEM images. TEM specimens were prepared using a focused ion beam with a microsampling function (FIB, Quanta3D, FEI, Hillsboro, OR, USA). The specimens were oxidized in an air furnace at 1273 K for 1.8 ks.

## 3. Results

### 3.1. Exfoliation Resistance, Fretting Wear, and Nanoindentation Hardness of Oxide Layers

**Figure 1:**
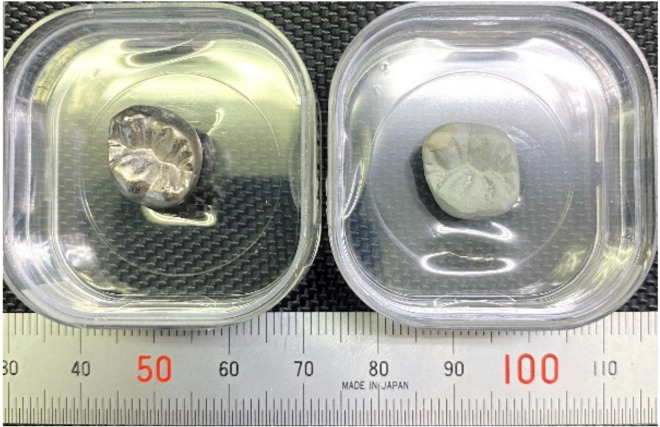
Prototype of titanium dental crown with/without oxidation. (**Left**) Ti crown without oxidation; (**Right**) with oxidation at 1273 K for 1.8 ks.

Based on previous reports [3,4], Ti–29Nb–13Ta–4.6Zr and Ti–36Nb–2Ta–3Zr–0.3O have a strong potential for use in dental prosthesis devices due to their color tone and microstructure. In addition, it has been also previously reported that the oxide layer on Ti–29Nb–13Ta–4.6Zr exhibits high exfoliation resistance [4]. Figure 1 shows a prototype of a titanium dental crown with/without oxidation. A smooth white oxide layer evenly coated the surface of a dental crown with complex shapes. Since such a uniform white layer was formed, fusing operations, such as a bonder and an opaque porcelain fusing operation of the metal bond porcelain crown, can be altered. This is expected to reduce the number of fusing operations.

**Figure 2:**
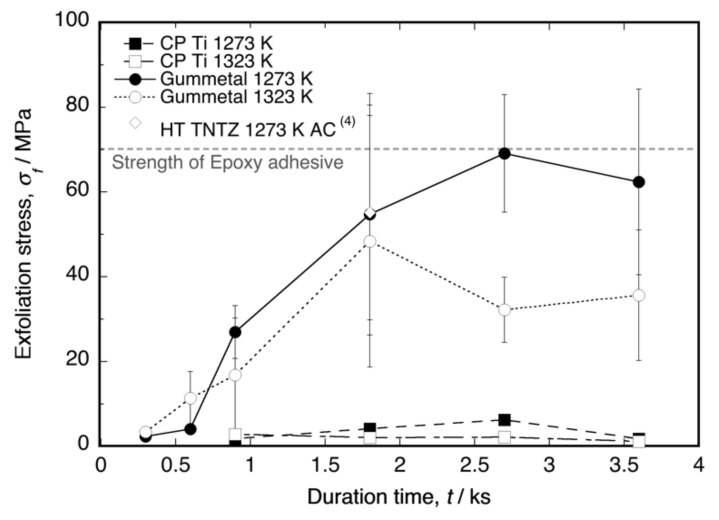
Exfoliation stress of oxidized CP Ti, Ti–36Nb–2Ta–3Zr–0.3O (Gummetal), and Ti–29Nb–13Ta–4.6Zr (TNTZ cooled in air after oxidation, data from [4]) as a function of the time duration at 1273 K and 1323 K. The error bar indicates standard deviation.

Figure 2 shows a plot of the exfoliation stress (*σ_f_*) as a function of the time duration. The *σ_f_* values of the oxide layers on Ti–36Nb–2Ta–3Zr–0.3O and Ti–29Nb–13Ta–4.6Zr [4] were clearly higher than that of the layer on CP Ti. The number of tests (*N*) per point ranged from three to four. The maximum stress in the Ti–Nb–Ta–Zr alloys was approximately the same as the maximum stress in the epoxy adhesives, i.e., approximately 70 MPa. According to the graph, *σ_f_* was dependent on the time duration, implying that a layer thickness exists for obtaining the maximum *σ_f_*. A comparison of the maximum *σ_f_* at each temperature with the layer thickness *l* in Ti–36Nb–2Ta–3Zr–0.3O has been reported [6], and on the basis of the results, the maximum *σ_f_* is likely to occur immediately before the maximum *l*. Therefore, our results suggest that *l* = 30–40 μm is the appropriate thickness for achieving maximum exfoliation resistance and a sufficiently high L*. In addition, a higher oxidation temperature tended to reduce *σ_f_*.

**Figure 3:**
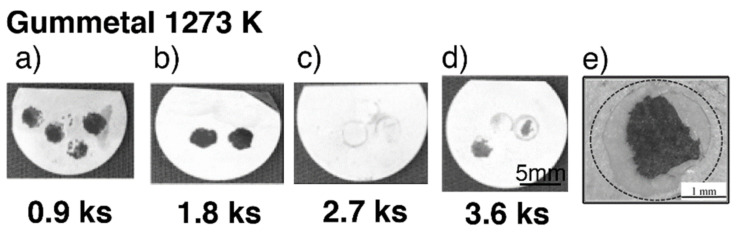
Exfoliated surface of specimens after exfoliation tests at each time duration in Ti–36Nb–2Ta–3Zr–0.3O. (**a**–**d**) Tested specimens after oxidization with different time durations (0.9–3.6 ks) at 1273 K; (**e**) enlarged image of a typical exfoliated surface.

**Figure 4:**
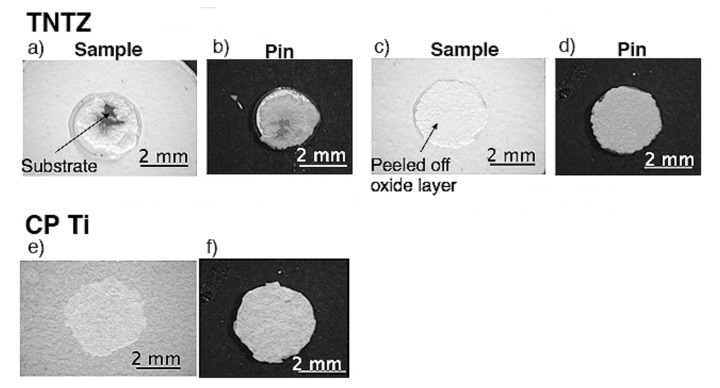
Exfoliated surface of oxidized specimens and stud pin after exfoliation tests. Oxidized condition was at 1273 K for 3.6 ks in air: (**a**–**d**) Ti–29Nb–13Ta–4.6Zr and (**e**,**f**) CP Ti. (**a**,**b**) *σ_f_* ≈ 70 MPa, (**c**,**d**) *σ_f_* ≈ 20 MPa, (**e**,**f**) *σ_f_* ≈ 7 MPa.

Typical exfoliated surface images are shown in Figure 3 and Figure 4. The exfoliated surfaces on Ti–36Nb–2Ta–3Zr–0.3O are mainly the result of interfacial failure, which means a dark-gray substrate was observed on the exfoliated surfaces after the test, except in the case shown in Figure 3c. Figure 3c is not an image of coherent failure; the epoxy adhesive was peeled off instead of the oxide layer because the exfoliation stress of the oxide was higher than that of the adhesive, as seen in Figure 2. Figure 4 shows an exfoliated surface and a counterface of the stud pin after the Ti–29Nb–13Ta–4.6Zr and CP Ti tests. An interfacial fracture occurred on Ti–29Nb–13Ta–4.6Zr as well as on Ti–36Nb–2Ta–3Zr–0.3O, while cohesion failure, such as that shown in Figure 4c,d, was rarely observed. In such cases, the *σ_f_* value decreased to approximately 20 MPa, though this *σ_f_* value was still much higher than that of CP Ti. Cohesion failure always occurred in the case of CP Ti; additionally, the *σ_f_* value was always less than 7 MPa.

**Figure 5:**
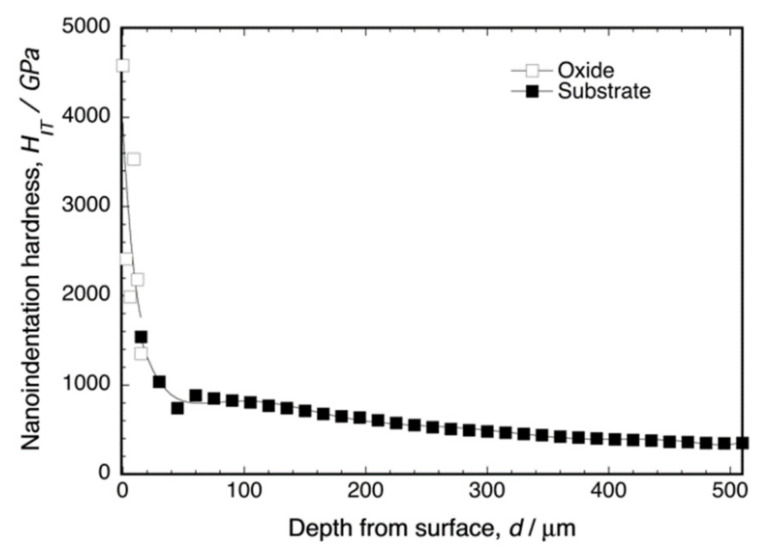
Nanoindentation hardness of oxidized Ti–29Nb–13Ta–4.6Zr at the interface. The oxidation conditions at 1273 K for 1.8 ks in air.

Figure 5 shows the depth profile of the nanoindentation hardness of the cross-section of oxidized Ti–29Nb–13Ta–4.6Zr at 1273 K for 1.8 ks. As shown in this graph, dense oxide exhibited very high hardness—*H*_IT_ ≥ 2000 for the oxide—whereas *H*_IT_ ≈ 320 for the substrate at a depth of 500 μm. Notably, the hardness in the oxide layer appeared to decrease gradually and continuously with increasing depth toward the substrate, and the hardness in the substrate also decreased gradually, which may be attributed to oxygen diffusion and phase transformation to the α + β lamellar structure. The microstructure cross-section is discussed in the subsequent section.

**Figure 6:**
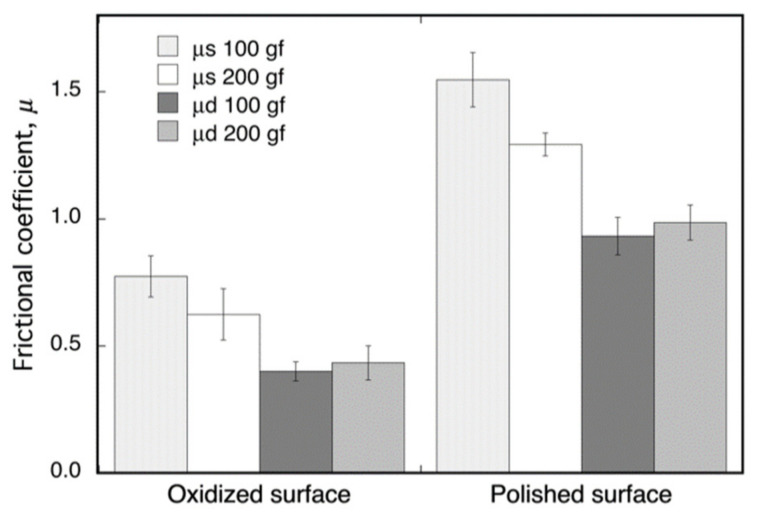
Dynamic (*μ*_d_) and static (*μ*_s_) frictional coefficients of oxidized and non-oxidized Ti–29Nb–13Ta–4.6Zr. The oxidation conditions at 1273 K for 1.8 ks in air.

The friction coefficients, *μ*, of the oxide surface on Ti–29Nb–13Ta–4.6Zr and of its mirror-polished metal surface, as measured by fretting friction, are shown in Figure 6. The oxidized Ti–29Nb–13Ta–4.6Zr was prepared by oxidation in an air furnace at 1273 K for 1.8 ks. Both the static and dynamic friction coefficients obviously decreased due to oxidation. This result suggests that an artificial tooth made of this material can potentially reduce friction against an involution tooth.

### 3.2. Results of Cross-Sectional SEM Observations and the Au Marker Method

**Figure 7:**
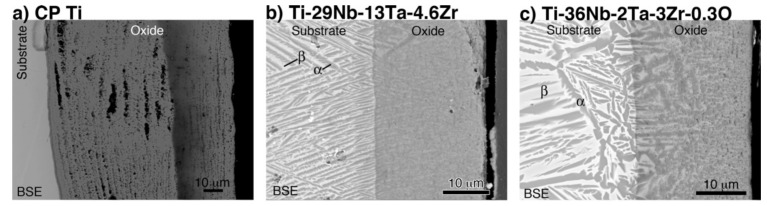
Back-scattered electron (BSE) images of the interfacial microstructure of oxidized (**a**) CP Ti, (**b**) Ti–29Nb–13Ta–4.6Zr, and (**c**) Ti–36Nb–2Ta–3Zr–0.3O. The oxidation conditions at 1273 K for 1.8 ks in air.

Cross-sectional back-scattered electron (BSE) images of oxidized CP Ti, Ti–29Nb–13Ta–4.6Zr, and Ti–36Nb–2Ta–3Zr–0.3O at 1273 K for 1.8 ks are shown in Figure 7. In Figure 7a, a porous microstructure with a stratified layer morphology, oxide particle layer, and one-by-one stacked layers with gaps are observed in the oxide layer on CP Ti. In contrast, as shown in Figure 7b,c, a dense oxide layer was formed on the Ti–29Nb–13Ta–4.6Zr and Ti–36Nb–2Ta–3Zr–0.3O substrates. As previously reported, the substrate microstructures beneath the oxides of Ti–29Nb–13Ta–4.6Zr and Ti–36Nb–2Ta–3Zr–0.3O exhibited lamellar structures that consisted of an α + β phase [3,4,6]. From the results of the electron probe microanalyzer (EPMA) mapping [6], Ti and O, and Nb and Ta, clearly separated in either the substrate or the oxide layer over the substrate. As mentioned in a previous report [6], because O is an α-phase stabilizer, the α-phase precipitated in the β-phase substrate due to O diffusion. The α-phase was observed beneath the oxide layer and along the grain boundary, which indicates that O diffused either interstitially or through larger defects, such as grain boundaries. With respect to the oxide layer in Ti–29Nb–13Ta–4.6Zr and Ti–36Nb–2Ta–3Zr–0.3O, the α + β microstructure was formed on the substrate.

**Figure 8:**
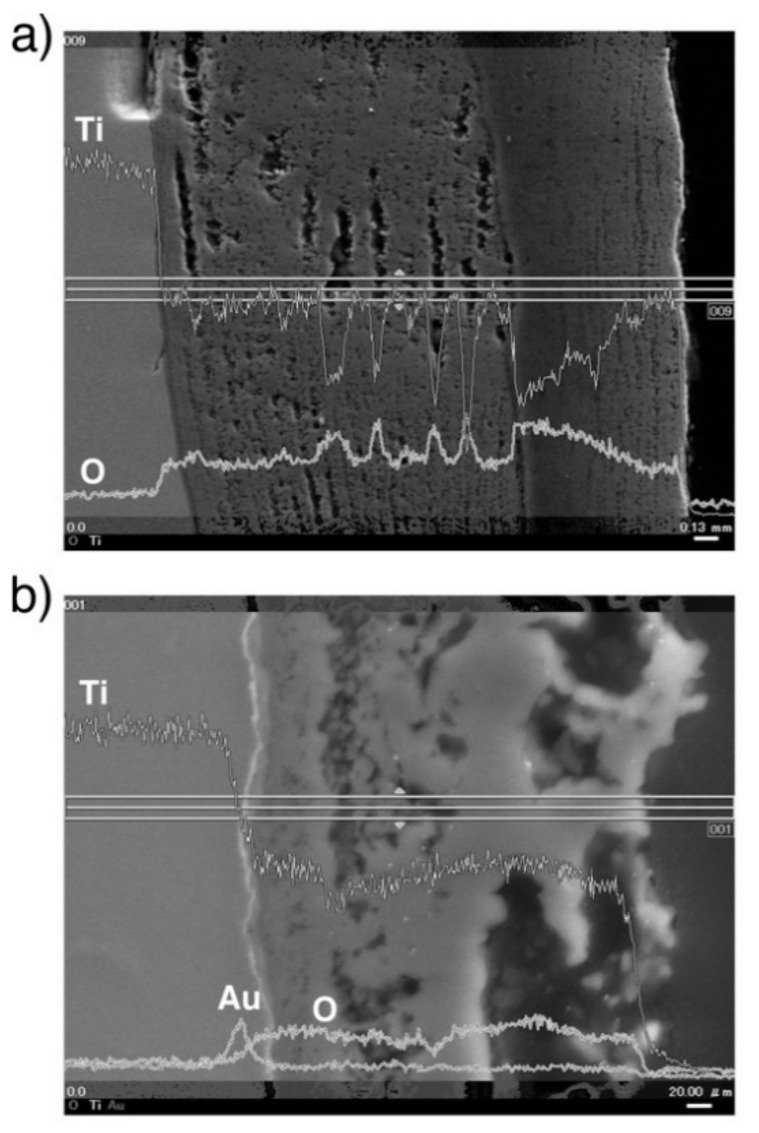
BSE image and line profiles of Au, O, and Ti of the cross-section at the interface of oxidized CP Ti (**a**) without marker insertion and (**b**) with Au marker insertion. The oxidation conditions at 1273 K for 1.8 ks in air.

**Figure 9:**
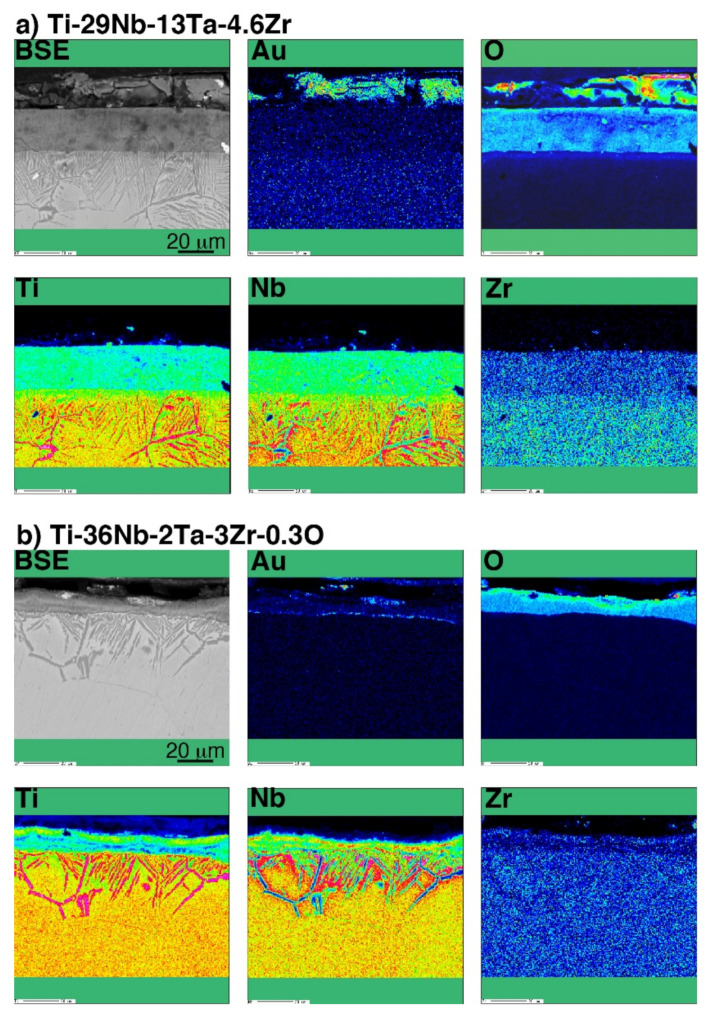
BSE image and chemical composition map of Au, O, Ti, Nb, and Zr in a cross-section of the interface of oxidized (**a**) Ti–29Nb–13Ta–4.6Zr and (**b**) Ti–36Nb–2Ta–3Zr–0.3O with Au marker insertion.

A line profile obtained by the SEM-EDS of oxidized CP Ti and Au-marked CP Ti is shown in Figure 8. As shown in Figure 8b, the Au marker insertion was distinguished by a thin bright line, which clearly differentiated between the Ti substrate and the oxide layer. This result is in good agreement with the results of degassing treatments on Ti with an Au marker [17,19]. In addition, the Au marker showed that the oxide layer growth was drastically suppressed. When the Au line was observed at the interface between the metallic substrate and the oxide layer, Ti out-diffusion was predominant during oxidation because Ti diffused through the surface and formed an oxide layer outside the marker. However, the results for Ti–29Nb–13Ta–4.6Zr and Ti–36Nb–2Ta–3Zr–0.3O are more complicated. The EPMA mapping results for Au-marked Ti–29Nb–13Ta–4.6Zr and Ti–36Nb–2Ta–3Zr–0.3O after oxidation are shown in Figure 9. Au was mostly detected on the top oxide surface in Ti–29Nb–13Ta–4.6Zr, as shown in Figure 9a, whereas it was detected at both the oxide surface and interface in Ti–36Nb–2Ta–3Zr–0.3O, as shown in Figure 9b. Notably, these are representative results, and other behaviors were also observed in other specimens of Ti–36Nb–2Ta–3Zr–0.3O, where the Au marker was observed only on the top surface, and vice versa. Therefore, these results suggest that both in and out-diffusion occurred during oxidation in these alloys.

### 3.3. Microstructural Observations by TEM

**Figure 10:**
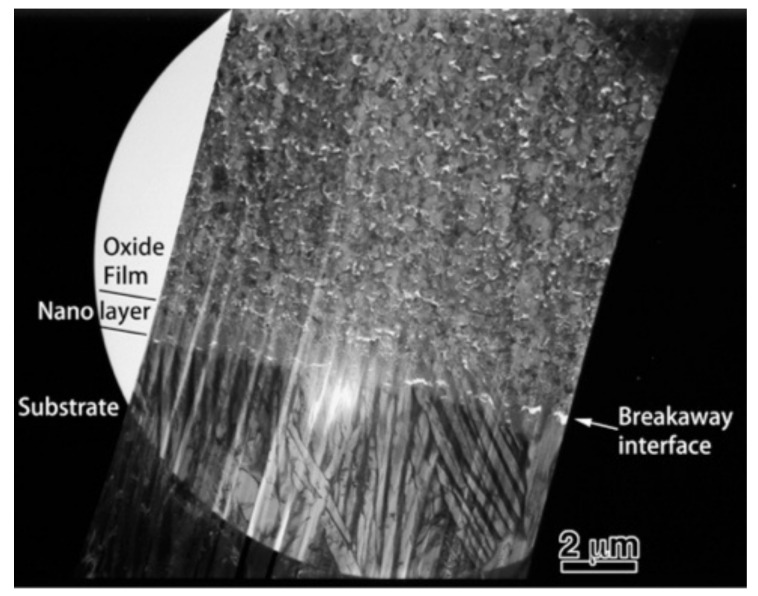
Low-magnification TEM image of a cross-section of oxidized Ti–29Nb–13Ta–4.6Zr. The oxidation conditions at 1273 K for 1.8 ks in air.

**Figure 11:**
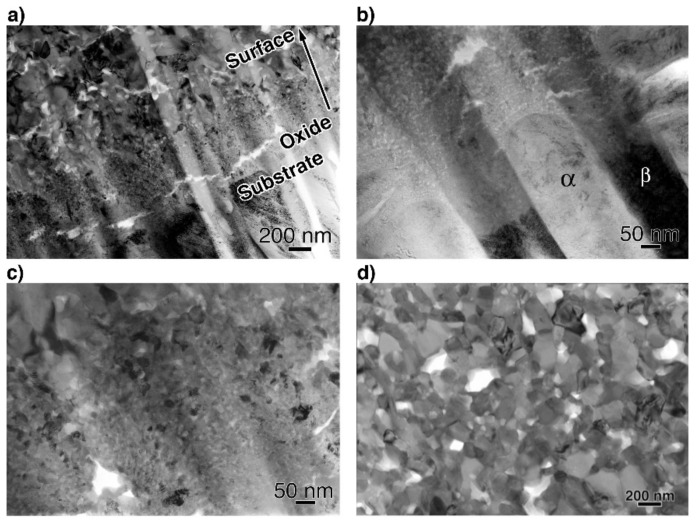
Bright-field TEM images of a cross-section (**a**,**b**) at the interface between the substrate and oxide layer, (**c**) at transition layer, and (**d**) at the oxide layer. The oxidation conditions at 1273 K for 1.8 ks in air.

To obtain more information related to the interfacial reaction, we observed cross-sections of the interface between the oxide and metal substrate in Ti–29Nb–13Ta–4.6Zr using TEM. A bright-field low-magnification image is shown in Figure 10. The interface consisted of the substrate, a transition layer with a thickness of 500–1000 nm, and a uniform oxide layer. Magnified images at the interface are shown in Figure 11a–c. The oxide layer appears to have formed continuously from the metallic substrate through the intermediary transition layer, as shown in Figure 11a. The enlarged images in Figure 11b,c reveal that the transition layer between the oxide layer and metallic substrate consists of nanosized grains, which correspond to the “nanolayer” in Figure 10. As shown in Figure 11c, increasing grain growth was observed within the nanolayer with increasing distance from the substrate, i.e., larger grains with diameters of several tens to approximately 100 nm appeared. Above the transition layer, equiaxed oxide grains, with an average grain size of approximately 200 nm, were homogeneously observed, as seen in Figure 11d. This result indicated that Mie scattering occurs within a visible light wavelength range since the particle size parameter (α=πδ/λ, δ: grain size; λ: wavelength) is α ~ 1 [20,21].

**Figure 12:**
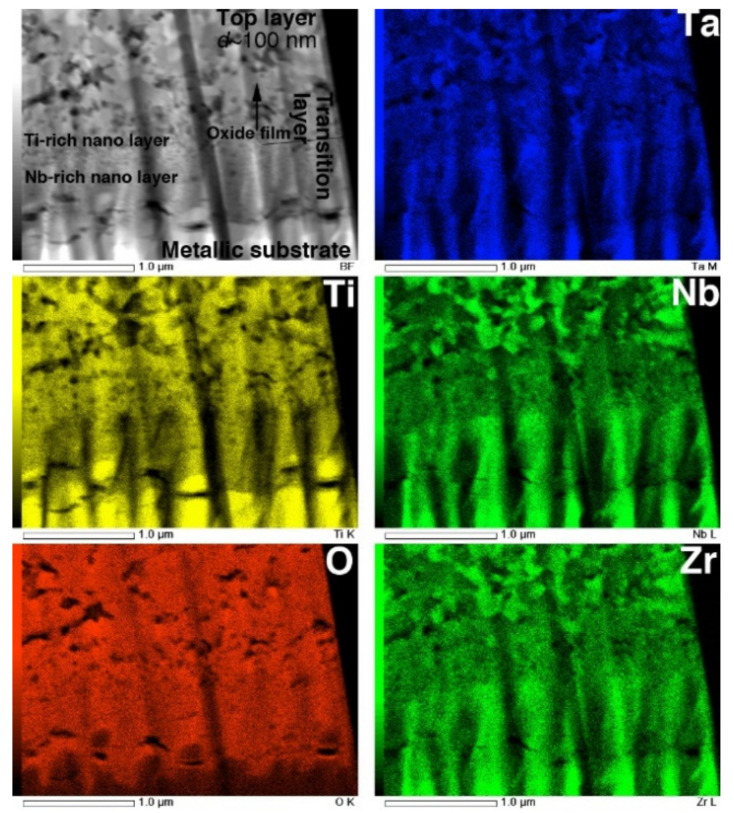
STEM image and EDS composition maps of Ti, O, Nb, Ta, and Zr in cross-section of interface of oxidized Ti–29Nb–13Ta–4.6Zr. The oxidation conditions at 1273 K for 1.8 ks in air.

The STEM and TEM-EDS mapping images of the interface are shown in Figure 12. In the O map, the boundary dividing the high- and low-O regions is clearly observed at the interface between the metallic substrate and the transition layer. The composition maps clearly reveal that the transition layer is composed of two layers consisting of Ti- and Nb-rich nanograins. This result corresponds to the interfacial transition layer observed in EPMA maps, reported previously [6]. Above the transition layers, the top layer consists of multiphase equiaxed oxides with diameters of approximately 100 nm, corresponding to the image in Figure 11c.

**Figure 13:**
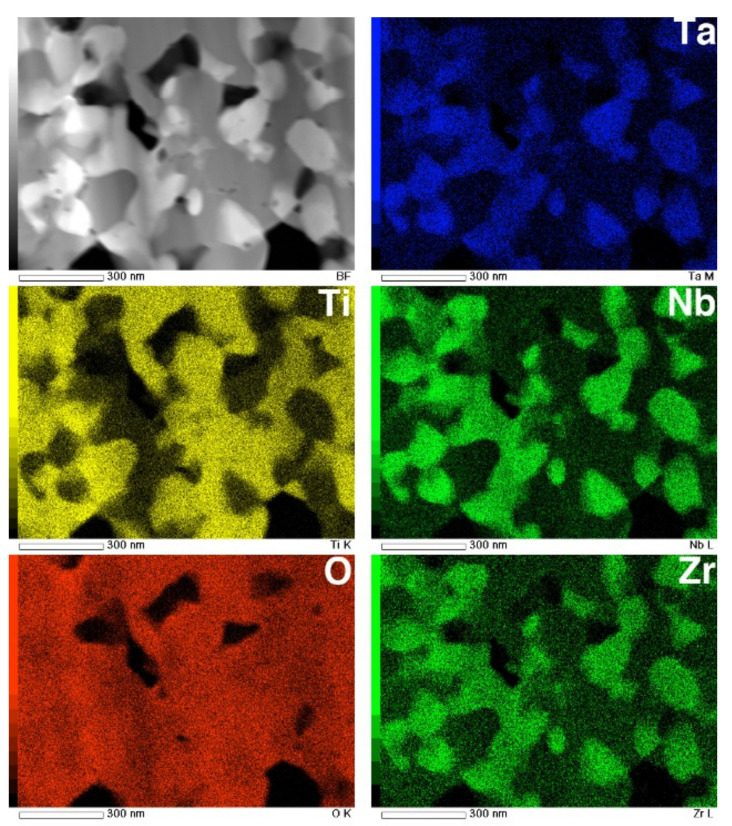
STEM image and EDS composition maps of Ti, O, Nb, Ta, and Zr in the cross-section of an oxide layer on Ti–29Nb–13Ta–4.6Zr. The oxidation conditions at 1273 K for 1.8 ks in air.

The STEM and EDS mapping images of an equiaxed oxide layer are shown in Figure 13. These composition maps suggest that the oxide layer consists of multiple phases. Phase separation into Ti-rich grains and Nb-, Ta-, and Zr-rich grains was identified. According to the XRD profile, the Ti-rich grains are likely TiO_2_, while the Nb- and Ta-rich grains are likely TiNb_2_O_7_ or Ti(Nb, Ta)_2_O_7_. Although Zr is generally recognized as being a neutral element with respect to β stabilization [2], the concentration of Zr atoms in the oxide-containing Nb and Ta was high compared to Ti-rich grains. These equiaxed submicron grains were homogeneously dispersed with TiO_2_ grains, and the grain diameters were 100–200 nm. These results indicate that both oxides grow almost simultaneously.

## 4. Discussion

### 4.1. Exfoliation Stress of Oxide-Coated Ti-Alloys

The results in Figure 2 and Figure 7 indicate that the high exfoliation resistance of Ti–36Nb–2Ta–3Zr–0.3O and Ti–29Nb–13Ta–4.6Zr is attributable to the dense and robust oxide microstructures and that the presence of fine particles and a composition-graded interfacial microstructure contribute to its high exfoliation resistance. In the case of CP Ti, a weak TiO_2_ layered structure broke prior to exfoliation at the interface. In addition, this high hardness of the oxide layer is suggested to result in a lower friction coefficient than that of the highly adhesive Ti-alloy metal surface.

Given the nanoindentation hardness results and the cross-sectional observations for Ti–29Nb–13Ta–4.6Zr in Figure 5 and Figure 7b, respectively, the interfacial failure was understandably predominant because the oxide layer was stronger than the interface between the substrate and oxide due to its dense and solid structure. A comparison between the *σ_t_* values of Ti–36Nb–2Ta–3Zr–0.3O and CP Ti indicates that the exfoliation stress of CP Ti is low and that its fracture is always due to a coherent failure. Thus, fracture always occurred inside the oxide layer, presumably because its multilayered microstructure consisted of a TiO_2_ monolayer and a gap. As the number of contacts among the oxide grains was much less than that in Ti–36Nb–2Ta–3Zr–0.3O, each oxide layer of Ti peeled off more easily than that of Ti–36Nb–2Ta–3Zr–0.3O; thus, the oxide layer of CP Ti was weaker than the interface.

Such a remarkable change in the exfoliation resistance due to the difference in the oxide structure was confirmed in our previous study [22], which investigated the effect of the Nb content on the oxide structure and exfoliation resistance of Ti-Nb alloys. Therefore, the apparent difference in the exfoliation stress of the oxide layer formed on the Ti-Nb-Ta-Zr alloy or CP Ti can be qualitatively attributed to the reasons mentioned in previous paragraphs.

The relationship between exfoliation stress and oxidation time duration shown in Figure 2, especially the increase in exfoliation stress with increasing oxidation duration in Gummetal, suggests a relationship with layer thickness. In our previous studies [4,6], the relationship between oxidation duration and oxide layer thickness was investigated. According to the results, the oxide layer thickness of both the CP Ti and Ti-Nb-Ta-Zr alloys increased with increasing time duration, and the respective thicknesses of TNTZ and Gummetal were approximately 12 and 25 μm at 1.8 ks and 18 and 36 μm at 2.7 ks. The brightness L* of TNTZ increased with increasing layer thickness; however, it almost saturated at approximately L* = 83 at 1.8 ks. On the other hand, the brightness of Gummetal gradually increased with increasing layer thickness until at least 3.6 ks and reached L* = 81 at 2.7 ks, which was the same level as that of TNTZ. In other words, the increase in exfoliation strength in Figure 2 is also considered to be related to the increase in oxide layer thickness.

### 4.2. Relationship between Oxidation Direction and Exfoliation Resistance in Terms of the Pilling–Bedworth Ratio

The results of cross-sectional observations of Au-marked specimens discussed in the previous section suggested clear differences between the oxidation processes of CP Ti and Ti–Nb–Ta–Zr alloys. In the case of CP Ti, because the Au marker was located at the interface, we concluded that out-diffusion by Ti through the Au marker was predominant during oxidation. On the contrary, the position of the Au marker indicated that both Ti diffusion to the surface and O diffusion into the substrate were the dominant processes during the oxidation of Ti–29Nb–13Ta–4.6Zr and Ti–36Nb–2Ta–3Zr–0.3O, i.e., not only in-diffusion of O but also the out-diffusion of Ti (and likely Nb, Ta, and Zr) played important roles during the oxidation of Ti–29Nb–13Ta–4.6Zr and Ti–36Nb–2Ta–3Zr–0.3O, whereas only out-diffusion was predominant in CP Ti. This difference between Ti and the Ti–Nb–Ta–Zr alloys is understandable because the alloying elements are mainly refractory metals with high melting points and because their diffusion rate is much lower than that of Ti or O. Additionally, the difference between the apparent growth rates and thicknesses of the oxide layers of CP Ti, Ti–29Nb–13Ta–4.6Zr [3,4], and Ti–36Nb–2Ta–3Zr–0.3O has been previously reported [6]. From the results, it is hypothesized that the slower diffusion rates of alloying elements, such as Nb, Ta, and Zr, affected the oxide formation mechanism, and that, as a consequence, the layer thickness and morphology changed. According to our previous study on the oxidation behavior of Ti-Nb alloys [22], the layer thickness growth rate of the oxide followed a parabolic function, and the activation energy *E* of the layer thickness growth obtained from it increased with increasing Nb content. The *E* value approached the *E* value of the diffusion of Ti and Nb in Ti-Nb from that of O in Ti. In addition, the improvement of exfoliation resistance in Ti-Nb alloys containing more than 13 mol% Nb was attributed to the densification of the oxide microstructure due to the phase separation caused by the addition of Nb beyond the dissolution limit of TiO_2_. Thus, the oxidation behavior of Ti-Nb binary alloys is similar to that of Ti-Nb-Ta-Zr alloys, suggesting that the similar oxidation behavior contributes to the high exfoliation resistance of the oxide layer in the Ti–29Nb–13Ta–4.6Zr and Ti-36Nb-2Ta-3Zr-0.3O alloys.

The Pilling–Bedworth ratio (PBR) is among the most important factors governing exfoliation between an oxide layer and a substrate [23]. The PBR is calculated by the following equation:

(1) $$(PBR)=V_{O}/V_{M}$$

*V_O_* and *V_M_* are the volume of the elementary cell of a metal oxide per ion and the corresponding metal per atom, respectively. When PBR ≈ 1, the volume difference is small; thus, an oxide forms continuously on a substrate. Consequently, compression stress is not generated at the interface of the oxide. If PBR < 1, the volume of the oxide is smaller than that of the metal; thus, a dense and continuous oxide microstructure cannot be obtained. Finally, in the case of PBR > 1, the volume of the oxide is greater than that of the metal; in this case, a continuous microstructure of the oxide is obtained; however, an interfacial fracture may tend to occur because compressive stress is introduced into the oxide layer. As an exception, when the oxide layer forms by out-diffusion, i.e., when Ti diffusion in the substrate toward the surface governs oxidation, the oxide layer forms on a free surface. Consequently, interfacial compression stress would not be produced during oxidation by out-diffusion, and in this case, exfoliation at the interface would not occur even if PBR > 1. However, in the case where the in-diffusion of oxide ions controls the formation of the oxide, new oxide forms at the interface between the metal and oxide. In this case, the PBR should be 1; otherwise, cracks would form at the interface due to the stress it generates.

The PBRs of the materials obtained in this study were estimated. It is assumed that the substrate could be either α- or β-Ti. The parameters used in this estimation were room-temperature parameters obtained from a database [24,25,26,27]. When TiO_2_ forms on the substrate, the PBR is 1.75; if the substrate β-Ti, the PBR is 1.77, while if TiNb_2_O_7_ forms on the α- or β-Ti, the PBR is 2.48 or 2.50, respectively. Therefore, in this study, with respect to the obtained oxide, all the PBRs were greater than 1; thus, a layer with a dense or continuous microstructure, but prone to exfoliation, was expected to be obtained.

In the case of CP Ti, the exfoliation stress was relatively low; however, our results indicated that this was attributable to its layered structure and not to interfacial compression stress due to the PBR being > 1. In addition, as previously mentioned, according to the results of Miura et al. [17,19] obtained using an Au marker method, TiO_2_ formation was controlled by out-diffusion. Therefore, the low exfoliation stress of TiO_2_/CP Ti resulting from the layered structure was weaker than the adherence of TiO_2_ to the metallic substrate.

Both Ti–29Nb–13Ta–4.6Zr and Ti–36Nb–2Ta–3Zr–0.3O exhibited a dense and continuous oxide layer. Although their PBR values were >1, corresponding to their dense robust morphology, their exfoliation stresses were very high. The results of the Au-marker experiments obviously indicated that in-diffusion contributed to oxide formation, resulting in the generation of interfacial stress that leads to interfacial fracture. In fact, the interface between the substrate and oxide was continuously connected, and cracks were hardly observed. Although an applied mechanics validation should be conducted to reach a determinate conclusion, the obtained results suggest that the hardness gradation structure at the interface in Figure 5 should contribute to reducing interfacial stress and, consequently, the development of strong exfoliation stress.

### 4.3. Oxide Layer Formation Mechanisms

As seen in Figure 12, the interface between the TNTZ alloy substrate and the oxide layer, i.e., the transition layer where the oxidation reaction proceeds, was divided into a Ta-rich nanocrystalline layer and an Nb-rich nanocrystalline layer on top of it. As shown in the EPMA elemental map in Figure 9, O diffusion from the substrate surface and into the substrate mainly along the grain boundary decreased the β stability in the area of increased O concentration, and the microstructure changed from a β-single structure to an α + β structure. It is clear from the results obtained in this study that the presence of alloying elements, especially Nb, has a significant effect on the oxide layer growth.

The addition of Nb to Ti reportedly increases oxidation resistance [22,28,29,30]. The following two reasons have been proposed for the suppression of oxidation by the addition of Nb to Ti: (1) reduction in O vacancies in the oxide by the principle of valence control; (2) Nb addition enhances the formation of the titanium nitride layer near the oxide/metal interface, which acts as a diffusion barrier and suppresses O diffusion. Ogawa and Miura-Fujiwara [22] concluded that the reduction in O vacancies in the oxide by (1) is more dominant than that by (2) in the densification of the TiO_2_ layer microstructure by Nb addition. Furthermore, in the case of Ti-Nb eutectic oxide precipitation, the densification is due to the suppression of outward diffusion of Ti by Nb at the reaction interface and the simultaneous redistribution of Nb into the TiO_2_ and TiNb_2_O_7_ phases during oxide precipitation.

**Figure 14:**
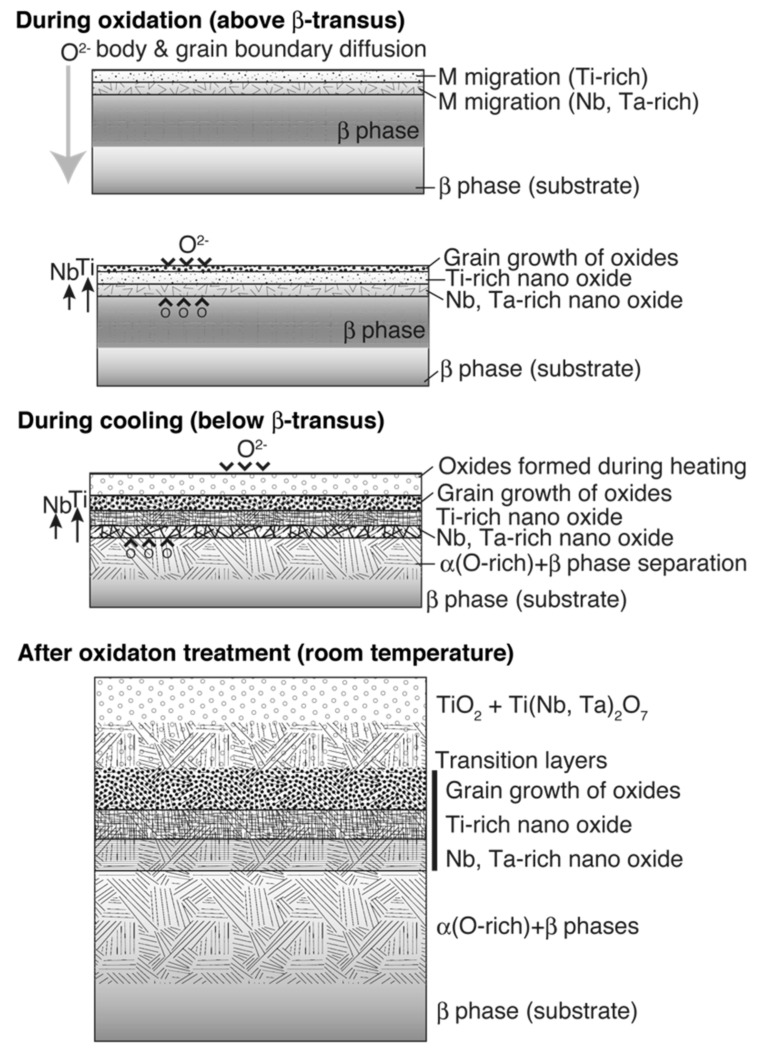
Illustrations of oxidation mechanisms for Ti–29Nb–13Ta–4.6Zr and Ti–36Nb–2Ta–3Zr–0.3O deduced from the obtained results.

Considering the results of previous reports [3,4,5,6,22] and those obtained in this study, the oxide layer formation mechanisms of CP Ti, Ti–29Nb–13Ta–4.6Zr, and Ti–36Nb–2Ta–3Zr–0.3O are discussed herein. Figure 14 is a schematic of oxide formation deduced from the results obtained for Ti–Nb–Ta–Zr alloys. Oxide layer formation on CP Ti was discussed in a previous paper [6]. As shown in this figure, oxide layer formation on Ti–Nb–Ta–Zr alloys can be explained as follows: as shown in Figure 14, during the oxidation treatment, partial α-stabilization occurred because O (=α stabilizer) diffused into the substrate, although the oxidation temperature was greater than the β-transus temperature (*T*_β_ < 1155 K). O diffusion was faster than oxide formation at the surface because Ti, Nb, and Ta and possibly Zr atoms migrated in the substrate, followed by phase transformation during oxidation to not only TiO_2_ but also Ti(Nb, Ta)_2_O_7_. According to the interfacial microstructure observed using TEM and EPMA, a fine Nb-rich oxide layer was formed under a Ti-rich oxide layer as the transition layer and oxide grains gradually grew within these transition layers. These results suggest that the rearrangement of Ti and other elements occurred within the transition layers because Nb diffusion is slower than Ti diffusion. Then, grain growth occurred through these transition layers. In addition, focusing on the oxide layer microstructure, as mentioned in the previous section, the α + β lamellar microstructure formed in the substrate was conserved in the portion of the oxide layer close to the interface, as evident from Figure 7b,c. This result indicates that long-range diffusion is suppressed during oxidation at the interface. This oxide growth mechanism probably resulted in the dense structured oxide layers of Ti–36Nb–2Ta–3Zr–0.3O and Ti–29Nb–13Ta–4.6Zr, and the oxide layer thickness becoming thinner than that of CP Ti can also be attributed to the mechanism [6]. When the temperature decreased to less than the β-transus temperature, α + β transformation occurred in the substrate prior to oxidation. Therefore, these results suggest that the phase separation of the oxide is the rate-controlling process in this mechanism, and that the differences in the apparent layer thickness and in the grain size between CP Ti and Ti–Nb–Ta–Zr alloys are attributable to the differences in their oxidation mechanisms.

## 5. Summary and Conclusions

In this study, the exfoliation resistance and oxide formation mechanism of Ti–36Nb–2Ta–3Zr–0.3O, Ti–29Nb–13Ta–4.6Zr, and CP Ti were investigated using an exfoliation test, a nanoindentation test, a fretting test, the Au marker method, and a cross-sectional microstructural observation of their oxidized specimens at 1273 K and 1323 K for 0.3–3.6 ks in air. The relationship between the exfoliation resistance and microstructure was discussed in addition to the oxide layer formation mechanism. The conclusions derived from this study are as follows:

1. The exfoliation stress, *σ_f_*, of the oxide layer on CP Ti is always <7 MPa, and its fracture is always caused by cohesion failure. The *σ_f_* of the oxide layer on Ti–36Nb–2Ta–3Zr–0.3O is much higher than that of the oxide layer on CP Ti, and the maximum *σ_f_* is approximately 70 MPa, which is approximately equal to the maximum stress of the epoxy adhesives. In the case of Ti–36Nb–2Ta–3Zr–0.3O, interfacial fracture occurs.
2. The high exfoliation resistances of Ti–36Nb–2Ta–3Zr–0.3O and Ti–29Nb–13Ta–4.6Zr are attributed to their dense and robust oxide microstructures; in addition, fine particles and a composition-graded interfacial microstructure may contribute to their high exfoliation resistance. In the case of CP Ti, its weak “piecrust-like” layered structure is broken prior to exfoliation at the interface.
3. The nanoindentation hardness of the oxide layers appears to decrease gradually and continuously with increasing depth toward the substrate; the hardness in the substrate also gradually decreases.
4. According to the results of the fretting frictional tests, both the static and dynamic friction coefficients obviously decrease as a consequence of the oxide layer formation.
5. The Au marker method indicates that out-diffusion plays a predominant role in oxide formation on CP Ti. Both in- and out-diffusion are involved in oxide formation on Ti–29Nb–13Ta–4.6Zr and Ti–36Nb–2Ta–3Zr–0.3O.
6. TEM observations of Ti−29Nb−13Ta−4.6Zr revealed that the transition layer consists of nanograins of a Ti-rich oxide phase and an Nb-, Ta-, and Zr-rich oxide phase at the interface between the oxide and the metal substrate. The oxide layer consists of dense submicron-sized TiNb_2_O_7_ and TiO_2_ grains, whereas the oxide layer on CP Ti consists of a layered structure with micron-sized TiO_2_ grains.
7. For Ti−29Nb−13Ta−4.6Zr and Ti−36Nb−2Ta−3Zr−0.3O, a robust oxidation layer with a multiphase equiaxed subnanomicrostructure forms continuously on the substrate through the nanostructured transition layer, and the high exfoliation resistance can be attributed to this strong and smooth graded structure.

## Data Availability

Data sharing is not applicable to this article.
